# Does Antithrombotic Therapy Affect Outcomes in Major Trauma Patients? A Retrospective Cohort Study from a Tertiary Trauma Centre

**DOI:** 10.3390/jcm11195764

**Published:** 2022-09-29

**Authors:** Stefano Sartini, Marzia Spadaro, Ombretta Cutuli, Luca Castellani, Marina Sartini, Maria Luisa Cristina, Paolo Canepa, Chiara Tognoni, Agnese Lo, Lorenzo Canata, Martina Rosso, Eleonora Arboscello

**Affiliations:** 1 Emergency Medicine Department, UOC MECAU, San Martino Policlinic University Hospital, Largo Rosanna Benzi 10, 16132 Genoa, Italy; 2 Department of Health Sciences, University of Genova, 16128 Genoa, Italy; 3 Hospital Hygiene Unit, Galliera Hospital, Via Alessandro Volta 8, 16128 Genoa, Italy; 4 Emergency Medicine Post-Graduate School, University of Genoa, Via Balbi 5, 16126 Genoa, Italy; 5 School of Medicine, University of Genoa, Via Balbi 5, 16126 Genoa, Italy

**Keywords:** trauma, coagulopathy, anticoagulants, antiplatelets, antithrombotic agents, bleeding, hemorrhage

## Abstract

Antithrombotic therapy may affect outcomes in major trauma but its role is not fully understood. We aimed to investigate adverse outcomes among those with and without antithrombotic treatment in major trauma. **Material and methods:** This is a retrospective study conducted at the Emergency Department (ED) of the University Hospital of Genoa, a tertiary trauma center, including all major trauma between January 2019 and December 2020. Adverse outcomes were reviewed among those without antithrombotic treatment (Group 0), on antiplatelet treatment (Group 1), and on anticoagulant treatment (Group 2). **Results:** We reviewed 349 electronic charts for full analysis. Group 0 were n = 310 (88.8%), Group 1 were n = 26 (7.4%), and Group 2 were n = 13 (3.7%). In-hospital death and ICU admission, respectively, were: n = 16 (5.6%) and n = 81 (26%) in Group 0, none and n = 6 (25%) in Group 1, and n = 2 (15.8%) and n = 4 (30.8%) in Group 2 (*p* = 0.123-*p* = 0.874). Altered INR (OR 5.2) and increasing D-dimer levels (AUC: 0.81) correlated to increased mortality. **Discussion:** Group 2 showed higher mortality than Group 0 and Group 1, however Group 2 had fewer active treatments. Of clotting factors, only altered INR and elevated D-dimer levels were significantly correlated to adverse outcomes. **Conclusions:** Anticoagulant but not antiplatelet treatment seems to produce the worst outcomes in major trauma.

## 1. Introduction

Trauma is a leading cause of mortality in upper-middle-income countries and the major cause of death and disability among young adults [1]. Trauma-induced coagulopathy (TIC) is present in 10–25% of major trauma patients and associated with 3–4 times increased mortality, and along with acidosis and hypothermia represents the “lethal triad” known since 1982 [2,3,4]. It is characterized by a derangement between coagulation and fibrinolysis processes and its management forms an integral part of hemostatic resuscitation in post-traumatic bleeding [5].

Massive transfusion guidelines were designed to address TIC; however, this condition can remain neglected when massive blood transfusion is not anticipated or properly managed [6]. Early identification and treatment of TIC are difficult and there is uncertainty regarding optimal therapeutic guidelines during the early phases of trauma resuscitation [7].

The aging population has resulted in a change in the demographics of trauma, with increased numbers of injured patients on antithrombotic agents [8]. Despite a better understanding of TIC in the general population, how antiplatelet (AP) and anticoagulant (AC) medications could affect coagulopathy and outcomes is not fully understood [9,10]. Early TIC has been suggested to result from a combination of inadequate thrombin generation, platelet dysfunction, fibrinogen depletion, and hyperfibrinolysis; therefore, medications that interfere with such mechanisms may alter platelets activation and coagulation cascades in cases of traumatic bleeding [11,12]. Most of the studies have been focused on traumatic brain injury, and they show contradictory results [13,14]. A timely reversal of anticoagulation in an acute setting can help to restore hemostatic functions and potentially reduce bleeding prior to definitive surgery or other interventions [15].

We aimed to study a major trauma population managed at our Emergency Department (ED), comparing adverse outcomes of those on AP, AC, or no antithrombotic treatment.

## 2. Materials and Methods

### 2.1. Study Design and Settings

This is a retrospective cohort study conducted at the ED of the University Hospital of Genoa, Italy, a tertiary trauma center and the main hub of the Liguria region for all specialties, with almost 125.000 hospital emergency admission per year. We reviewed all electronic charts of major trauma patients admitted to our ED between January 2019 and December 2020.

### 2.2. Participants and Data Collection

Inclusion criteria were age > 16 years old and access to our hospital with the criteria of major trauma.

We defined major trauma if any of the following was present: altered physiology (SBP < 90, GCS < 13, SatO2 < 90 despite oxygen, RR < 10 or >22); need for advanced life support in the pre-hospital setting or in another hospital (intubation/supraglottic aids, decompression/drainage/fluids > 2000 mL); altered anatomy (thorax contusion and respiratory distress or flailed chest, head–neck–thorax–abdomen-penetrating wounds, facial injury with airways compromission, signs of basal skull fracture, suspected unstable hip fracture, open limb fracture, burns > 30%, airways compromission); dangerous mechanism of injury (fall from height >3 m, prolonged vehicle extrication, a death on scene, car/motorbike/train collision with walker/biker, high-speed impact, injury from blast).

Exclusion criteria were age <16, death before ED arrival, pregnancy, not enough clinical or laboratory data, and minor trauma.

We investigated all enrolled patients for: age, gender, vitals parameter at ED admission, clinical history, antithrombotic treatment in place, clotting tests (PT, aPTT, fibrinogen, D-dimer), full-body CT-scan evidence, and source of active bleeding (cerebral, thoracic, abdominal, pelvis, limbs), treatment (open surgery, endovascular, conservative), need of blood transfusions within 24 h from hospital admission, and the department where the patients were admitted (Trauma Center, ICU, ED).

We divided the population into three groups: Group 0 (those not on any antithrombotic treatment), Group 1 (those on AP), and Group 2 (those on AC), and we compared their baseline variables and adverse outcomes. The three groups shared identical inclusion and exclusion criteria.

As per our internal major trauma management protocol, we administered tranexamic acid (1 gr plus 1 gr in 6–8 h) to all suspected- or confirmed-bleeding patients, and gave reversal agents (idarucizumab for those on dabigatran and four-factors prothrombin complex concentrate (50 IU/Kg) for the others) to patients on oral AC treatment.

In our hospital, a major bleeding transfusion protocol is not yet in place; however, at least two units of packed red blood cell 0 negative are available in the ED for all unstable patients. Fresh frozen plasma, platelets, fibrinogen, and cryoprecipitate are available for transfusionat at our blood bank; however, these are given based on clinical judgement.

We conducted this study in accordance with the ethical principles of the Declaration of Helsinki and approval by our institution (Ospedale Policlinico San Martino, Genoa, Italy).

### 2.3. Outcomes Measures

We considered as clinical adverse outcomes: in-hospital mortality, ICU admission, or new hospital admission within 30 days from discharge.

### 2.4. Data Analysis and Statistical Methods

We entered patients’ data on an ad hoc-created database in Excel (Microsoft^®^). We conducted a descriptive analysis of the data.

We presented patients’ characteristics as median and interquartile ranges (IQR) for continuous variables and expressed as absolute values along with percentages for categorical variables. As the data did not display a normal distribution, we evaluated every possible numerical transformation of the data. As none of these was able to reduce the effect of skewness, we analyzed the data by means of non-parametric tests.

We compared all variables among the three sub-populations (on AP, on AC, or not on AP or AC) with and without adverse outcomes. For the comparison, we used Kruskal–Wallis for continuous data and χ2 test or Fisher’s exact test in case of non-continuous variables.

We made clotting factors analysis between survivors and non-survivors, and other than with median and IQR, we dichotomized data as altered and non-altered using following normal value as per our laboratory reference range: platelets 130–430 × 10⁹ cells/L, INR 0.80–1.20, aPTT 28–40 seconds, and fibrinogen 2–4 gr/L. We found it more clinically meaningful in addition to the crude statistical analysis significance.

We used stepwise logistic regression models and significance levels for removal from the model *p* > 0.05 to estimate the odds ratios (ORs) and 95% confidence intervals (CI) to identify which altered clotting factors were the best predictor of in-hospital mortality. We included all the significant variables in the univariate analysis in the model. Specifically, we entered clotting factors (platelets, fibrinogen, aPTT, INR) as binomial variables (altered parameter 1, not altered 0). We entered age into the model as a non-removable variable, although it was not significant given the importance of this parameter in the risk of death.

We used univariate receiver operating characteristic (ROC) analysis to find the best cut-off for D-dimer values to predict in-hospital mortality. We used the Youden index to calculate the best cut-off (max (Sensitivity+Specificity-1)). Consequently, we used the best cut-off D-dimer value to calculate the incidence rate of mortality.

All tests were two-sided, and we considered a *p* value less than 0.05 as statistically significant. We included all participants for whom the variables of interest were available in the final analysis without imputing missing data. All statistical analyses were performed with Stata/SE 14.2 (StataCorp, College Station, TX, USA).

## 3. Results

### 3.1. Characteristic of Study Subjects

Of the 703 electronic charts reviewed, only 349 were included for full analysis as 196 did not meet inclusion criteria for major trauma, and 158 were excluded for lacking data.

Overall, 256 (73.3%) were male with a median age of 51 (32–66 IQR). The quantity of those undergoing a direct admission from scene to our ED was 316 (90.5%), whilst 33 (9.4%) came after first being evaluation in other hospitals. Group 0 (no antithrombotic agents) contained 310, Group 1 (on AP) contained 26, and Group 2 (on AC) contained 13. Population general characteristics are reported in Table 1.

### 3.2. Descriptive Data

In Group 1, 16 were on aspirin, 9 on clopidogrel, and 1 on dual antiplatelet (aspirin + clopidogrel). Of these, nobody died, and 16 were admitted to ICU with full recovery.

In Group 2, two patients were on edoxaban, three on apixaban, two on dabigatran, four on warfarin, and one was not reported. Of these, the two who died were both on warfarin, and those admitted to ICU were one on edoxaban, one on apixaban, and two on warfarin. Reversal agents were given to all bleeding patients as appropriate.

Full-body CT scan with evidence of active bleeding were n = 125 (40.32%) of Group 0; n = 10 (38.46%) of Group 1; and n = 5 (38.46%) of Group 2. Specifically, the cerebral bleeding quantity was 62 (20.00%) in Group 0, 5 (19.23%) in Group 1, and 4 (30.77%) in Group 2; the quantity of patients with haemo-pneumothorax and/or cardiac tamponade was 14 (4.52%) in Group 0 and 2 (15.38%) in Group 1; the quantity of patients with abdominal bleeding was 24 (7.74%) in Group 0 and 1 (3.85%) in Group 1; the quantity of patients with pelvic bleeding was 13 (4.19%) in Group 0, 1 (3.85%) in Group 1, and 1 (7.69%) in Group 2; last, the number of patients with limbs displaying significant bleeding was 12 (3.87%) in Group 0 and 1 (3.85%) in Group 1.

Looking at antithrombotic agents and active bleeding, we found that of the ten patients in Group 1, seven were on aspirin, two were on clopidogrel, and one was on dual antiplatelets (aspirin + clopidogrel); of the five in Group 2, one was on edoxaban, one was on dabigatran, and three were on warfarin.

Overall, 79 (22.64%) were discharged directly from ED after the appropriate observation period, and of those admitted to hospital, 45 (16.67%) went to a specialized Trauma Center ward. No delayed bleeding or new hospital admissions within 30 days from discharge were observed in any group.

A total of 36 patients received blood products, and of these, 12 had >2 units of packed red blood cells for hemotransfusion.

### 3.3. Main Results

Table 2 show the number of patients with altered clotting factors values at ED admission (comparing survivors and non-survivors in each group). Group 1 is not reported in this table as all patients on AP survived.

Stepwise logistic regression analysis about clotting factors, adjusted for age and type of treatment chosen, was performed to assess predictors of in-hospital mortality. Only age and altered INR were found to be significantly correlated with an increased likelihood of death. (Table 3).

**Figure 1:**
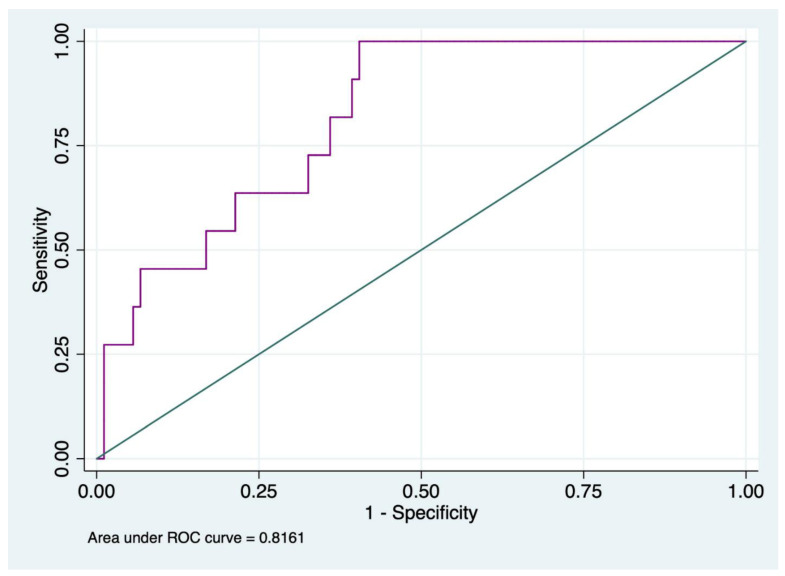
ROC curve analysis of D-dimer values to assess in-hospital mortality.

D-dimer was analyzed separately as only 5% of all patients had a D-dimer lower than 700. ROC analysis to predict in-hospital mortality for D-dimer is shown in Figure 1.

The ROC analysis showed that the best cut-off value of D-Dimer related to in-hospital mortality was ≥11,961, with a sensitivity of 100% and specificity of 59.55%, and the AUC was 0.82 (IC95% 0.71–0.92).

The incidence rates of in-hospital mortality for D-dimer values ≥ 11,961 ng/mL were 9.66 out of 1000 patients.

## 4. Discussion

In this retrospective cohort study, we evaluated the adverse outcomes of major trauma patients at a tertiary university hospital trauma center among patients with and without antithrombotic treatment.

In-hospital mortality was higher in Group 3 (15.3%) than in Group 1 (5.6%) and Group 2 (none); however, active bleeding was similar in all three groups (38–40%). The incidences of death and bleeding in our study are in line with previous findings [16,17]. Although anticoagulated patients had higher death rates, bleeding did not differ between the three groups. The increased risk of bleeding after trauma among those on direct oral anticoagulants is a matter of debate; however, in our cohort, bleeding was not increased in the anticoagulated group [9,18,19].

Group 1 and 2 were older than Group 0 (73–76.3 vs. 48 median age, respectively) but the ISS and vital parameters at ED presentation did not differ. Even if age differed among the three groups, it was an independent factor related to in-hospital death (OR 1.04, 95% CI, 1.00–1.07). This may have affected the decision to actively treat these patients. It is known that elderly trauma patients have poorer outcomes than the younger ones, mainly due to physiological changes, comorbidities, nutritional deficits, and pre-medication [20,21,22]. Nonetheless, no one in Group 1 died, which could be related to the small sample of the group.

Group 2 had the higher rates of intracranial bleeding (30.77% vs. 20.00% and 19.23% in Group 0 and 1, respectively) and conservative management (92.31% vs. 62.26% and 76.92% in Group 0 and 1, respectively). It has been reported that TIC is present in 22.7–60% after traumatic brain injury according to previous studies, and it is correlated to the severity of the injury [23,24]. Moreover, elderly patients trauma with intracranial bleeding are more prone to develop TIC, as Takayama et al. found [25]. Furthermore, conservative management is the treatment of choice in geriatric trauma and in those on AC, reducing the opportunity for effective intervention in this setting of patients [21,26].

In our population, we had no delayed bleeding or differences in those on AT, even in those onclopidogrel, even if predisposed to it [27]. Of the five patients on AC with active bleeding, 3/5 were on warfarin. From previous studies, new direct oral anticoagulants have shown reduced risks of major bleeding in AC patients with respect to those on vitamin-K antagonists but an increased risk on mortality was confirmed only for those on AP plus AC [28,29].

Looking at clotting factors, we found that in Group 0, although all were statistically different, they increased INR and reduced fibrinogen median values out of the normal range in non-survivors. Of these two, only the former was significantly altered among survivors and non-survivors. In Group 2, only increased INR was significantly higher in non-survivors, and all of them had an altered INR (Table 2).

This was confirmed by the stepwise logistic regression analysis, which showed that only altered INR, other than age, was significantly related to in-hospital mortality, with an OR of 5.203 (95% CI 1.262–21.459) (Table 3).

D-dimer was increased in the majority of patients and ROC curve analysis showed that a value ≥ 12.000 ng/mL had an AUC of 0.81, predicting an incidence rate of in-hospital mortality of 9.84/1000. Initial elevated D-dimer has been found as correlated to poor outcomes (massive bleeding and death) in many studies as it represents the magnitude of tissue damage reflecting precocious hyperfibrinolysis [30,31]. Furthermore, Ishii et al. found that not only D-dimer but also PT-INR were significantly related to increased mortality in blunt trauma, reporting an AUC of 0.86 and 0.83, respectively [32], and these findings are in line with our results. Conversely from what McQuilten et al. found, in our study, fibrinogen was not the only predictor of in-hospital mortality [33]. Even if it is well known that fibrinogen’s consumption is a central mechanism of TIC, its role as a predictor of mortality is still a matter for debate [34,35].

## 5. Limitations

There are several limitations to this study. First, the retrospective nature of the study did not allow for the full availability of the data, and 158 patients had to be excluded for missing information. Second, our ED received fewer major trauma cases than expected in 2020 because lockdown for COVID-19 caused a significant reduction in trauma admissions. Consequently, Group 1 and 2 were small, and they may not represent the whole population. Finally, viscoelastic parameters were not available, so we could not verify how antithrombotic agents may affect them.

## 6. Conclusions

Those on anticoagulants but not on antiplatelet agents were more exposed to adverse outcomes when affected by major trauma compared with those not on such a treatment. However, this result may be related to other factors, such as age and the decision for conservative management other than coagulopathy. Altered INR and increased D-dimer were correlated to higher mortality, irrespective of pre-injury antithrombotic medication. Thus, the development of TIC may not be affected by antithrombotic agents. Further analysis is needed with a larger population and with viscoelastic parameters to verify whether, and in which measures, TIC can be influenced by antithrombotic treatment.

## Figures and Tables

**Table 1:** Cohort demographics and clinical characteristics.

	Group 0 No Antithrombotic Agents (N = 310)	Group 1 on Antiplatelets (N = 26)	Group 2 on Anticoagulants (N = 13)	*p*-Value
**Age, years**	48 (30–61)	76 (64–81)	73 (70–81)	<0.001
**Male**	229 (73.9%)	19(73.1%)	8 (61.5%)	0.575
**Direct admission** **from scene**	281 (90.6%)	23 (88.4%)	12 (92.3%)	0.193
	**Vital Parameters**			
**Systolic blood pressure, mmHg**	130 (118–140)	145 (125–157)	130 (110–134)	0.0524.
**Dyastolic blood pressure, mmHg**	80 (70–81)	80 (70–90)	75 (69–80)	0.2699
**SatO2 (%)**	98 (96–99)	97 (94–98)	98 (96–98)	0.0631
**Heart rate, per minute**	80 (74–94)	77.5 (70–85)	78 (70–84)	0.0480
**Respiratory rate, per minute**	15 (14–17.5)	19 (18–21)	14	0.0910
**Temperature, Celsius**	36.5 (36–36.6)	36.5 (36–36.7)	36.2 (35–36.5)	0.3568
	**Laboratory Results**			
**White cell count,** 10⁹ cells/L	12.85 (9.7–17.2)	11.17 (7.6–14.7)	13.5 (9.4–16.9)	0.1547
**Haemoglobin,** g/L	14.2 (12.8–15.2)	13.5 (12.1–15.0)	12.2 (11.6–13.5)	<0.5
**Platelets,** 10⁹ cells/L	234 (193–274)	227 (186–279)	214 (178–283)	0.4308
**INR**	1.09 (1.03–1.16)	1.00 (1–1.13)	1.29 (1.19–2.76)	<0.001
**aPTT,** seconds	29.2 (26.9–31.7)	28.8 (26.4–31.9)	34.9 (29.4–47.1)	<0.05
**Fibrinogen,** g/L	2.59 (2.10–2.96)	3.7 (3.53–4.28)	2.9 (2.27–3.68)	<0.01
**D-dimer,** ng/mL	11435 (2351–27,090)	4774 (2252–13,356)	1450	0.2707
**Troponin,** μg/L	0.015 (0.015–0.015) [0.11–1.32]	0.15 (0.015–0.015) [0.015–0.043]	0.015 (0.015–0.02) [0.015–0.093]	0.5588
**Creatinin,** mg/dL	0.9 (0.8–1)	0.9 (0.7–1.2)	0.9 (0.8–1.2)	0.6067
**C-reactive protein,** μg/dL	2.9 (2.9–3.6)	2.95 (2.9–9.4)	3.3 (2.9–7.4)	0.2752
	**Trauma Characteristics and Management**			
**Altered physiology**	63 (20.3%)	4 (15.4%)	2 (15.4%)	0.894
**Altered anatomy**	21 (6.8%)	2 (7.7%)	1 (7.7%)	0.977
**Dangerous mechanism** **of injury**	297 (95.8%)	21 (80.8%)	12 (92.3%)	<0.05
**ISS**	24.5 (15.8–37.2)	21.2 (18.2–28.9)	22.1 (14.2–31.8)	0.12
**Active bleeding**	125 (40.3%)	10 (38.5%)	5 (38.5%)	0.975
**Surgical treatment**	111 (35.8%)	5 (19.2%)	1 (7.7%)	<0.05
**Endovascular treatment**	16 (5.2%)	2 (7.7%)	0	0.687
**Conservative treatment**	193 (62.3%)	20 (76.9%)	12 (92.31%)	<0.05
**Lenght of stay**	7.9(2–16)	12.4 (2–23.9)	9.61 (4.68–11.49)	0.6469
	**Outcomes**			
**In-hospital death**	16 (5.2%)	0	2 (15.4%)	0.129
**ICU admission**	81 (26.1%)	6 (23.1%)	4 (30.8%)	0.874

Data are presented as median (interquartile range: 25°–75° P) and only for troponin (range); otherwise, count (%) as appropriate. Abbreviations: INR = international normalized ratio; ICU = Intensive Care Unit; ISS = injury severity score.

**Table 2:** (**1**) Clotting factors median and 25–75% IQR at admission and mortality in Group 0 and Group 2 (Group 1 values can be found in Table 1 as no deaths were found in this group). (**2**) Number of patients with altered clotting factors at admission and mortality in group 0 and group 2.

(**1**)						
	***Group 0***			***Group 2***		
	**Survivors**	**Non-Survivors**	***p*** **-Value**	**Survivors**	**Non-Survivors**	***p*** **-Value**
*Platelets* (10⁹ cells/L) *n.v. 130*–*430*	234 (196–274)	179 (139–227)	<0.01	236(175–301)	150 (145–155)	0.1671
*INR* *n.v. 0.80* *–* *1.20*	1.08 (1.03–1.15)	1.35 (1.11–1.51)	<0.001	1.12 (1.08–2.42)	4.94 (4.57–5.32)	<0.05
*aPTT (seconds)* *n.v. 28* *–* *40*	29 (26.8–31.6)	30.7 (28.9–33)	<0.05	32(27.6–47.1)	42.15 (34.9–49.4)	0.4298
*Fibrinogen (g/L)* *n.v. 2.0* *–* *4.0*	2.60 (2.23–3.05)	1.81 (1.12–2.35)	<0.01	3.68 (2.27–3.88)	2.22 (1.55–2.90)	0.2482
(**2**)						
	***Group 0***			***Group 2***		
	**Survivors**	**Non-Survivors**	***p*** **-Value**	**Survivors**	**Non-Survivors**	***p*** **-Value**
*Altered PLT*	11 (3.7%)	2 (13.3%)	0.072	1 (9.1%)	0	0.657
*Altered INR*	40 (13.8%)	9 (60%)	<0.01	7 (63.64%)	2 (100%)	0.305
*Altered aPTT*	108 (38%)	4 (26.7%)	0.376	6 (54.5%)	1 (50%)	0.906
*Altered Fibrinogen*	32 (25.8%)	4 (50%)	0.136	0 (-)	1 (50%)	0.171

(**1**) Abbreviations: INR = international normalized ratio; n.v. = normal values. (**2**) Data are presented as number of patients count and %. Abbreviations: PLT: platelets; INR = International Normalized Ratio.

**Table 3:** Logistic regression analysis of clotting factors to predict in-hospital mortality.

	Odds Ratio	St. Error	z	p > [z]	95% Conf. Interval	
**Age**	1.038	0.019	2.06	0.039	1.002	1.076
**INR**	5.203	3.761	2.28	0.023	1.262	21.459

## Data Availability

The data presented in this study are openly available in our database (repository: Stefano Sartini, UOC MECAU, Ospedale Policlinico San Martino, Genova).

## References

[1] World Health Organization (2018). Global Health Estimates 2016: Deaths by Cause, Age, Sex, by Country and by Region, 2000–2016.

[2] Callcut R.A., Kornblith L.Z., Conroy A.S., Robles A.J., Meizoso J.P., Namias N., Meyer D., Haymaker A., Truitt M.S., Agrawal V. (2019). The why and how our trauma patients die: A prospective Multicenter Western Trauma Association study. J. Trauma Acute Care Surg..

[3] Moore E.E., Moore H.B., Kornblith L.Z., Neal M.D., Hoffman M., Mutch N.J., Schöchl H., Hunt B.J., Sauaia A. (2021). Trauma-induced coagulopathy. Nat. Rev. Dis. Prim..

[4] Van P.Y., Holcomb J.B., Schreiber M.A. (2017). Novel concepts for damage control resuscitation in trauma. Curr. Opin. Crit. Care.

[5] Kashuk J.L., Moore E.E., Millikan J.S., Moore J.B. (1982). Major Abdominal Vascular Trauma—A Unified Approach. J. Trauma Inj. Infect. Crit. Care.

[6] Spahn D.R., Bouillon B., Cerny V., Duranteau J., Filipescu D., Hunt B.J., Komadina R., Maegele M., Nardi G., Riddez L. (2019). The European guideline on management of major bleeding and coagulopathy following trauma: Fifth edition. Crit. Care.

[7] Maegele M., Schöchl H., Cohen M.J. (2014). An Update on the Coagulopathy of Trauma. Shock.

[8] Reske-Nielsen C., Medzon R. (2016). Geriatric Trauma. Emerg. Med. Clin. N. Am..

[9] Bläsius F.M., Laubach M., Andruszkow H., Lübke C., Lichte P., Lefering R., Hildebrand F., Horst K. (2021). Impact of anticoagulation and antiplatelet drugs on surgery rates and mortality in trauma patients. Sci. Rep..

[10] Daley M.J., Trust M.D., Peterson E.J., Luftman K., Miller A.H., Ali S., Clark A., Aydelotte J.D., Coopwood T.B., Brown C.V. (2016). Thromboelastography Does Not Detect Preinjury Antiplatelet Therapy in Acute Trauma Patients. Am. Surg..

[11] Park M.S., Spears G.M., Bailey K.R., Xue A., Ferrara M.J., Headlee A., Dhillon S.K., Jenkins D.H., Zietlow S.P., Harmsen W.S. (2017). Thrombin generation profiles as predictors of symptomatic venous thromboembolism after trauma: A prospective cohort study. J. Trauma Acute Care Surg..

[12] Floccard B., Rugeri L., Faure A., Denis M.S., Boyle E.M., Peguet O., Levrat A., Guillaume C., Marcotte G., Vulliez A. (2012). Early coagulopathy in trauma patients: An on-scene and hospital admission study. Injury.

[13] Savioli G., Ceresa I., Luzzi S., Lucifero A.G., Di Marco M.P., Manzoni F., Preda L., Ricevuti G., Bressan M. (2021). Mild Head Trauma: Is Antiplatelet Therapy a Risk Factor for Hemorrhagic Complications?. Medicina.

[14] Barmparas G., Kobayashi L., Dhillon N.K., Patel K.A., Ley E.J., Coimbra R., Margulies D.R. (2019). The risk of delayed intracranial hemorrhage with direct acting oral anticoagulants after trauma: A two-center study. Am. J. Surg..

[15] Coimbra R., Hoyt D.B., Anjaria D.J., Potenza B.M., Fortlage D., Hollingsworth-Fridlund P. (2005). Reversal of Anticoagulation in Trauma: A North American Survey on Clinical Practices among Trauma Surgeons. J. Trauma Inj. Infect. Crit. Care.

[16] Kauvar D.S., Lefering R., Wade C.E. (2006). Impact of Hemorrhage on Trauma Outcome: An Overview of Epidemiology, Clinical Presentations, and Therapeutic Considerations. J. Trauma Inj. Infect. Crit. Care.

[17] Capizzi A., Woo J., Verduzco-Gutierrez M. (2020). Traumatic Brain Injury: An Overview of Epidemiology, Pathophysiology, and Medical Management. Med. Clin. N. Am..

[18] Santing J.A., Lee M.Y.X., van der Naalt J., Brand C.L.V.D., Jellema K. (2022). Mild Traumatic Brain Injury in Elderly Patients Receiving Direct Oral Anticoagulants: A Systematic Review and Meta-Analysis. J. Neurotrauma.

[19] Fuller G., Sabir L., Evans R., Bradbury D., Kuczawski M., Mason S.M. (2020). Risk of significant traumatic brain injury in adults with minor head injury taking direct oral anticoagulants: A cohort study and updated meta-analysis. Emerg. Med. J..

[20] Hashmi A., Ibrahim-Zada I., Rhee P., Aziz H., Fain M.J., Friese R.S., Joseph B. (2014). Predictors of mortality in geriatric trauma patients: A systematic review and meta-analysis. J. Trauma Acute Care Surg..

[21] Hildebrand F., Pape H.-C., Horst K., Andruszkow H., Kobbe P., Simon T.-P., Marx G., Schürholz T. (2016). Impact of age on the clinical outcomes of major trauma. Eur. J. Trauma Emerg. Surg..

[22] Boltz M.M., Podany A.B., Hollenbeak C.S., Armen S.B. (2015). Injuries and outcomes associated with traumatic falls in the elderly population on oral anticoagulant therapy. Injury.

[23] Wafaisade A., Dgu T.R.O., Lefering R., Tjardes T., Wutzler S., Simanski C., Paffrath T., Fischer P., Bouillon B., Maegele M. (2010). Acute Coagulopathy in Isolated Blunt Traumatic Brain Injury. Neurocritical Care.

[24] Maegele M. (2013). Coagulopathy after traumatic brain injury: Incidence, pathogenesis, and treatment options. Transfusion.

[25] Takayama W., Endo A., Koguchi H., Murata K., Otomo Y. (2020). Age-related differences in the impact of coagulopathy in patients with isolated traumatic brain injury: An observational cohort study. J. Trauma Acute Care Surg..

[26] Llompart-Pou J.A., Pérez-Bárcena J., Chico-Fernández M., Sánchez-Casado M., Raurich J.M. (2017). Severe trauma in the geriatric population. World J. Crit. Care Med..

[27] Colombo G., Bonzi M., Fiorelli E., Jachetti A., Bozzano V., Casazza G., Solbiati M., Costantino G. (2021). Incidence of delayed bleeding in patients on antiplatelet therapy after mild traumatic brain injury: A systematic review and meta-analysis. Scand. J. Trauma Resusc. Emerg. Med..

[28] Antoni A., Schwendenwein E., Binder H., Schauperl M., Datler P., Hajdu S. (2019). Delayed Intracranial Hemorrhage in Patients with Head Trauma and Antithrombotic Therapy. J. Clin. Med..

[29] Rønning P., Helseth E., Skaansar O., Tverdal C., Andelic N., Bhatnagar R., Melberg M., Skaga N.O., Aarhus M., Halvorsen S. (2021). Impact of Preinjury Antithrombotic Therapy on 30–Day Mortality in Older Patients Hospitalized with Traumatic Brain Injury (TBI). Front. Neurol..

[30] Hayakawa M., Maekawa K., Kushimoto S., Kato H., Sasaki J., Ogura H., Matauoka T., Uejima T., Morimura N., Ishikura H. (2016). High D-Dimer Levels Predict a Poor Outcome in Patients with Severe Trauma, Even with High Fibrinogen Levels on Arrival. Shock.

[31] Zhang J., He M., Song Y., Xu J. (2018). Prognostic role of D-dimer level upon admission in patients with traumatic brain injury. Medicine.

[32] Ishii K., Kinoshita T., Kiridume K., Watanabe A., Yamakawa K., Nakao S., Fujimi S., Matsuoka T. (2019). Impact of initial coagulation and fibrinolytic markers on mortality in patients with severe blunt trauma: A multicentre retrospective observational study. Scand. J. Trauma Resusc. Emerg. Med..

[33] McQuilten Z.K., Wood E.M., Bailey M., Cameron P.A., Cooper D.J. (2016). Fibrinogen is an independent predictor of mortality in major trauma patients: A five-year statewide cohort study. Injury.

[34] Rourke C., Curry N., Khan S., Taylor R., Raza I., Davenport R., Stanworth S., Brohi K. (2012). Fibrinogen levels during trauma hemorrhage, response to replacement therapy, and association with patient outcomes. J. Thromb. Haemost..

[35] Fries D., Martini W.Z. (2010). Role of fibrinogen in trauma-induced coagulopathy. Br. J. Anaesth..

